# Digital Health Solutions for Indigenous Mental Well-Being

**DOI:** 10.1007/s11920-019-1056-6

**Published:** 2019-07-01

**Authors:** Jennifer M. Hensel, Katherine Ellard, Mark Koltek, Gabrielle Wilson, Jitender Sareen

**Affiliations:** Department of Psychiatry, University of Manitoba, 771 Bannatyne Ave, Winnipeg, MB Canada

**Keywords:** Indigenous, Digital health, Mental health, Telemental health, Social media

## Abstract

**Purpose of Review:**

This review summarizes digital health solutions being used for Indigenous mental well-being, with emphasis on available evidence and examples reported in the literature. We also describe our own local experience with a rural telemental health service for Indigenous youth and discuss the unique opportunities and challenges.

**Recent Findings:**

Digital health solutions can be grouped into three main categories: (1) remote access to specialists, (2) building and supporting local capacity, and (3) patient-directed interventions. Limited evidence exists for the majority of digital solutions specifically in Indigenous contexts, although examples and pilot projects have been described. Telemental health has the strongest evidence, along with a growing evidence for web-based applications, largely led by Australia. Other digital approaches remain areas of promise requiring additional study. Co-design and service integration and respect for Indigenous history and ideologies are essential for success.

**Summary:**

While the use of digital health solutions for Indigenous mental well-being holds promise, there is a limited evidence base for most of them. Future efforts to expand the use of digital solutions in this population should adhere to best practices for the delivery of Indigenous health services.

## Introduction

Internationally, health systems are making significant investments in digital health for health promotion, facilitating self-management, and improving access to care [1]. Digital health solutions, also referred to as “virtual healthcare” and sometimes “electronic or e-Healthcare”, may involve any form of information technology to enhance healthcare. Digital health solutions for mental well-being are one of the fastest growing uses [2]. In regions with high Indigenous representation, like Canada and Australia, use of digital solutions has specifically been recommended to address mental health needs and gaps in services [3, 4]. The significant health inequities experienced by the Indigenous have resulted from the complex interplay between geography, socioeconomic determinants of health, differing cultural conceptualizations of health, and longstanding effects of colonialism, intergenerational trauma, and structural violence leading to reinforcement of stigma and mistrust of Western health care [5•, 6]. The mental health needs among the Indigenous are often significant and unique [7, 8], with the potential to benefit substantially from digital health solutions, especially those developed and implemented in a culturally informed way.

In this review, we summarize the use of digital health solutions for mental well-being in Indigenous populations. We also discuss our own Rural Northern Telehealth Service (RNTS), which provides remote mental health services to Indigenous youth in Manitoba, Canada.

## Summary of Digital Health Solutions

We have grouped digital solutions into three categories: (1) remote access to specialists, (2) building and supporting local capacity, and (3) patient-directed interventions. Most of these digital approaches in psychiatry have been previously reviewed [9, 10], so here we focus on their application specifically in Indigenous populations. We used an inclusive search term for ‘Indigenous,’ developed in consultation with a local librarian (see Table 1), and focused on literature from Australia, USA, Canada, and New Zealand, developed regions with high Indigenous representation. This is not an exhaustive review, and there is a general dearth of rigourous intervention research conducted in Indigenous populations [11]. We have focused on recent studies and examples of digital solutions currently being used in Indigenous contexts (see Table 2 for summary).

**Table 1:** Indigenous search term developed in consultation with a librarian with expertise in Indigenous literature

Indigenous search term	
Indigenous OR Aboriginal OR “First Nations” OR Metis OR Inuit* OR Ojib* OR Anishinaabe OR Anishinabe OR Anishnabe OR Cree OR Dene OR “Alaska* native” OR “native Alaska” OR “native American” OR Mohawk OR Maori OR Nunavut OR Nunavik OR “Torres Strait Island*” OR Koori OR Goori OR Murri OR Nyoongah OR Koorie OR Yolngu OR Anangu OR Palawa OR Nunga OR Ngarrindjeri OR “Murray Island” OR “Mer Island”

**Table 2:** Summary of the available digital health solutions for Indigenous mental well-being with examples, strengths, and limitations

Digital solution	State of the evidence	Examples	Benefits	Limitations
*Remote access to specialists*				
Telemental health	Moderate to Strong • Quantitative studies demonstrating feasibility and cost effectiveness • Qualitative studies demonstrating acceptability	• Psychiatric assessment and management • Psychotherapy	• Reduces need for travel and has associated cost saving benefits • Keeps individuals in their home community • Facilitates involvement of family and other supports	• Some prefer face-to-face interactions, and may feel it depersonalizes the ‘human connection’ • May detract from local recruitment and capacity building efforts
Electronic consult	Fair • Pilot study in Indigenous Inuit community found cost-savings and provider satisfaction	• ChamplainBASE Nunavut project	• Keeps individuals in their home community • Enhances continuity of care • Enhances primary care provider satisfaction • Reduces need for travel and has associated cost saving benefits	• Relies on primary care assessment; specialist provides advice only • Requires access to a secure platform for information exchange
*Building and supporting local capacity*				
Electronic learning	Poor • No specific studies in Indigenous contexts	• Health care provider courses • Mental Health First Aid Training	• Standardizes material for large groups • Allows for distribution of information over large geographical distances • Enhances attendance rates	• Lack of face-to-face interaction, which is preferred by some • Lack of supervision may lead to underdeveloped skills • Difficulties assessing whether acquired skills are being utilized in practice
Electronic screening and decision support tools	Fair • Positive results in subset analyses and pilot studies	• YouthCHAT • Anchorage Depression Management DSTs • Intimate Partner Violence DSTs	• May help reduce racialized discrimination • Assists in identifying risky behaviors and health concerns • Facilitates patient-centred discussion surrounding treatment options	• Requires the health care provider knows how to work with the decision output
Virtual communities of practice	Poor • No specific studies in Indigenous contexts	• ECHO Ontario First Nations, Inuit, Metis Program	• Promotes collaborative learning among health professionals • Allows for team members to remain in their home community • Increased participant self-efficacy and satisfaction with reduced isolation	• Attendance may be limited by competing demands • Learning models utilized may not be accepted by all participants • Requires access to a multi-user digital platform
*Patient-directed interventions*				
Web-based applications	Fair to Moderate • Small number of RCTs demonstrating evidence for specific apps • Studies limited to Australian contexts	• Mindspot • ibobbly • AIMhi Stay Strong	• Tailored to the specific needs of a targeted group • Increases accessibility in a cost-effective, portable manner • Potential for co-design and adaptation of existing applications to be culturally relevant	• Variable accessibility dependent on the user, the environment, and the app design itself • Variable uptake by health care workers, due to their level of tech support, workload levels, office policies, and perceptions among staff members
Digital storytelling	Fair • Some preliminary evidence of benefit in Indigenous Alaskan youth	• Digital storytelling as a component of suicide prevention kits in Indigenous Alaskan Youth	• Enhances the sharing of personal experiences and learning from peers • May promote stronger intergenerational bonds • Allows a medium for users to reflect back on their own experiences and identify one’s own coping strategies	• Issues surrounding confidentiality • May perpetuate stereotypes • Can trigger past traumas among some members
Social media	Poor • No specific studies in Indigenous mental health • Some positive evidence of behavior change in other domains	• Indigenous run Twitter and Facebook accounts (Ex. @IndigenousX) • Social marketing campaigns for lifestyle changes	• Provides a platform for users to showcase their own unique identity • Allows for the sharing of information and ideas among millions of users • May enhance community connections • Ability to be tailored to user’s culture • Allows users to gain a sense of power/control	• May or may not be moderated leading to potential negative and/or harmful content • Accessibility and uptake will vary by user, and availability of technology

## Remote Access to Specialists

Given the high proportion of Indigenous people living in underserved rural areas, remote access to specialists is frequently required. Two modalities for remote specialist access are (1) telemental health and (2) electronic (e-)consult.

### Telemental Health

Telemental health has been used for decades and involves the practitioner and the care recipient interacting by videoconferencing [12]. Telemental healthcare can include psychiatric evaluations, psychotherapy, psychoeducation, and medication management to a range of treatment settings [12]. A review of telemental health services for Indigenous Australians identified the potential to reduce the disparity in the health status of Indigenous rural populations and achieve cost savings from reduction of travel time and greater access to specialist care [13••]. Other noted benefits included the social and emotional well-being benefits of receiving care in one’s own community, reduced feelings of alienation, and the facilitation of family attendance during care [13••].

In a study on the perspectives among Indigenous community members towards telemental health in Ontario, Canada, Gibson et al. [14] reported a diversity of perspectives, not unlike what has been identified among non-Indigenous recipients of telemental healthcare [15]. The need not to travel was a benefit to some, while others identified that getting out of the community could be therapeutic. Additionally, the notion that telemental health depersonalized the human connection that is embedded in many Indigenous cultures was raised as a concern. Conversely, there were respondents who felt they could be more forthcoming over video which they viewed as less intimidating. In another study about provider likelihood to offer telepsychotherapy to Indigenous communities, the most important consideration was perceived usefulness for the individual care recipient, a finding the authors suggested could overcome perceived and actual cultural barriers [16]. In these studies, the concern arose that remote services through telemental health could detract from local capacity building—which some suggested could be costlier. It was suggested that novel uses for the technology could go ‘outside of the box’, beyond the classic patient-provider interaction, to meet the needs of the community in a culturally informed way [14].

### Electronic (e-)Consult

In recent years, indirect consultation, traditionally done in hallways or over the telephone, has expanded to the digital realm. Store and forward and electronic (e-)consult involve asynchronous transmission of messages between healthcare professionals to avoid a formal referral [9]. The potential for e-consult has been described for psychiatry, highlighting rural providers as a receptive group given geographical barriers and long wait times for psychiatric consultation [17]. Psychiatry e-consults may be less likely to avoid a referral than other specialty areas and may have more uses for continuity of care [17, 18].

An example of e-consult within an Indigenous setting is the e-consult pilot conducted in Nunavut, Canada, where the population is predominantly Inuit [19]. Evaluation of this pilot over nearly 2 years demonstrated improved access to specialists and improved provider experience of care, with an estimated cost savings of $1100.93 per e-consult through avoided referrals and costs of travel and lost wages [19]. E-consult is emerging as a viable digital solution for specialist advice; however, for psychiatry, it is especially recommended that it be integrated with other services along a continuum of care [17].

## Building and Supporting Local Capacity

Building local capacity through task shifting involves training and supporting local providers or lay individuals to enhance knowledge or develop skills that they might not otherwise have within their current roles. Although some conflicting evidence exists for the effectiveness of task shifting and high provider workloads can be a barrier [20], task-shifting is a promising approach to increase service delivery in resource constrained environments that have unique needs. Potential challenges could arise around confidentiality in small communities, with varying preferences between novel approaches and more conventional channels for mental healthcare [21]. Digital technology can facilitate task shifting through electronic learning (e-learning), electronic screening and decision support tools (DSTs), and continued collaboration and supervision within virtual communities of practice [20, 22].

### E-Learning

E-learning is an increasingly popular method of education delivery to enable flexible accessibility to standardized materials and improve cost effectiveness [23]. In a recent systematic review, e-learning was as effective as traditional learning approaches among undergraduate healthcare professionals [23]. E-learning has been adopted in mental health, including examples in emergency health providers in rural settings with increased self-efficacy reported among participants [24], and for suicide prevention and Mental Health First Aid training resulting in increased attendance of individuals in both urban and rural settings [25, 26]. Mental Health First Aid has been adapted specifically to Indigenous communities [27]; however we could not locate any examples of e-learning modules for Indigenous Mental Health First Aid. One noted pitfall of e-learning for Mental Health First Aid is the absence of face-to-face interaction among group members, with recent literature proposing a blend of both face-to-face training and e-learning [26]. Additional challenges include particular topics or learner characteristics that are more amenable to e-learning vs in person learning [20, 23], and potential lack of supervision which may lead to underdeveloped skills not being translated into practice [28]. E-learning tailored to Indigenous mental health represents one area of future study.

### Electronic Screening and Decision Support Tools

Screening and DSTs offer an efficient way to promote assessment and collaborative treatment decisions. YouthCHAT, adapted from an adult-based electronic screening tool for mental wellbeing, is being used in New Zealand primary care settings with high Indigenous Maori attendance to identify risky behavior and mental health concerns [29]. The tool was adapted with Indigenous input including making it accessible in the Maori language. DSTs are often electronic tools that help patients clarify their values and preferences related to the decisional conflict and support patient involvement in their healthcare. A project collaboration between primary care and behavioral health services in Anchorage, USA, created a depression management DST to improve depression care for Alaskan Native and American Indian individuals [30]. A pilot study of the iPad-based DST demonstrated positive benefits for providers and patients which facilitated discussion around individualized treatment plans. In a separate randomized controlled trial of a web-based DST for intimate partner violence in New Zealand [31], 27% of the study population was Indigenous Maori. A subgroup analysis found significantly higher reductions in the primary outcomes—depressive symptoms and intimate partner violence exposure—among the Indigenous participants compared to the non-Indigenous participants. The authors postulated that this finding may be related to the tool’s ability to contradict a normalized cultural perspective on intimate partner violence among Indigenous women, and to overcome racialized discrimination in healthcare.

### Virtual Communities of Practice

Communities of practice promote collaborative learning among healthcare professionals who come together at regular time intervals to share knowledge surrounding a particular subject**.** Historically, these meetings would occur in person, but technology has now allowed these communities to transcend geography [22]. One example is Project ECHO (Extension for Community Healthcare Outcomes), a collaborative model of medical education and care management that connects rural primary care teams with specialist teams for regular case-based sessions held virtually using multipoint video conferencing [22]. Evaluation of a Project ECHO mental health in Ontario, Canada, reported increased participant self-efficacy, high satisfaction, and reduced isolation [32]. This group has developed a Project ECHO that focuses on Indigenous well-being (https://camh.echoontario.ca/first-nations-inuit-metis-wellness/). The 20-session curriculum, open to registration from across the province, covers a variety of topics related to Indigenous wellness, of which many have a mental health component. The specialist team consists of a psychiatrist, social work, and an Indigenous Elder among others. Novel approaches like this can enhance bidirectional learning and provide a community of practice for often isolated healthcare providers.

## Patient Directed Digital Solutions

### Web-Based Applications

Apps targeting mental wellbeing are disrupting the self-management space, making resources more accessible, cost-effective, and portable to the user [33••]. Apps are ideally co-designed to meet the needs of the user group, which usually translates to improved uptake and benefit, although few Indigenous-specific mental health apps have been rigourously studied. MindSpot is a multicomponent service in Australia offering a live virtual assessment with a psychologist and access to guided web-based self-management courses. Courses were modified to contain Indigenous content, and an evaluation comparing Indigenous and non-Indigenous users found comparable improvements for both groups [34]. Similarly, a pilot-randomized controlled trial of ibobbly, a suicide prevention app for Indigenous youth in Australia, found significant reductions in depression and anxiety symptoms in app users compared to waitlist controls, but no change in suicidal ideation [35]. ‘AIMhi Stay Strong’, also from Australia, was initially a therapist supported brief therapy intervention that was converted to an electronic format. The intervention utilizes cognitive behavioral principles to identify behaviors that are either helping or hindering mental health, set goals, build support, and work towards changing behaviors in a positive way. The app-based format incorporates Indigenous content and imagery for use by healthcare providers and their Indigenous clients [36••].

In a qualitative study of Indigenous Australians who had used AIMhi Stay Strong or ibobbly, Povey et al. [33••] identified three overarching themes regarding App acceptability: (1) user characteristics, (2) environmental characteristics, and (3) app design. Personal characteristics included illness factors, literacy and language, interest in technology, and the influence of a particular individual’s historical factors. Environmental factors included community awareness, stigma, and the integration of the app with other supports and care pathways (e.g., emergency services). App design was critical; the app had to be considered attractive to the user and meet their needs, with appropriate information security. User engagement was enhanced when information was delivered through culturally relevant language, with a purposeful journey ending in resolution [33••]. In a related study of the barriers and enablers of e-health adoption among Indigenous healthcare workers [36••], barriers were not substantially different from those identified in healthcare more broadly—a lack of technology support, demanding workloads, procurement and practice polices, and negative staff perceptions. Future studies will help to inform how these tools can best be implemented to be effective solutions for Indigenous populations.

### Digital Storytelling

Digital storytelling represents a unique and creative approach in the field of mental health. The objective of digital storytelling is to capture one’s story through the combination of first-person narratives, music, poetry, photography, and video, in a short digital clip made accessible to viewers. Digital storytelling promotes openness to share personal experiences and can encourage and support viewers to reflect back on their own life experiences, identify personal coping strategies, and to learn from their peers [37].

Digital storytelling as an intervention for Indigenous Alaskan youth was deemed a useful tool in suicide prevention kits, thought to facilitate stronger, protective intergenerational bonds, and used as a “reminder” to the Indigenous youth for “key reasons for living” [38]. While many benefits of digital storytelling have been identified, some pitfalls include accessibility issues, confidentiality, the risk of perpetuating stereotypes, and the potential triggering of past traumas [37]. The emphasis of digital story telling on the individual within his or her community and past [38] lends itself well to Indigenous healing philosophies. With limited research to date, this is an area that should be further explored.

### Social Media

Use of social media—virtual communities and networks that share information and ideas—among Indigenous youth and young adults is widespread. Findings from Australia suggest that use of Facebook might actually be higher among the Indigenous population than the general population [39••]. In a literature review on social media use among Indigenous young people, Rice et al. [39••] describe several drivers of use. These include a way to showcase one’s Indigenous identity, gain a sense of power and control, use of multimedia which aligns with the orally and visually focused culture, and community connections. Noted health-related applications of social media are health promotion and social marketing campaigns for behavior change. While not specifically for mental health, successful social marketing campaigns have been launched for healthy lifestyle change [40] and smoking cessation [41]. Lastly, Twitter—the microblogging platform—has been discussed as a venue for Indigenous perspectives to be shared, and for communities to lead social innovation and enhance social and emotional well-being [42]. One example is @IndigenousX, an Indigenous run Twitter and Facebook account [43]. Social media is an emerging medium that will undoubtedly continue to be harnessed in novel ways for advancement of health and well-being [44].

## Local Experience: The Rural Northern Telehealth Service

Since 2010, the Rural Northern Telehealth Service (RNTS) has provided multidisciplinary mental health support for youth living in underserved Indigenous communities throughout Manitoba, with a combination of telemental health and itinerant visits. Our experience with the development and expansion of this service has revealed important factors for success in the provision of virtual care in Indigenous communities.

## The Manitoba Context

The province of Manitoba is a prairie province centrally located in Canada. It encompasses 650,000 km^2^, 57% of which is boreal forest, known as “The Canadian Shield.” The population is approximately 1.3 million, with almost half residing rurally. Indigenous peoples comprise 18% of the provincial population [45], the highest and fastest growing population rate in the country. Over 60,000 Indigenous persons live on 63 reservations, many of which are remote and isolated without all season road access. The Indigenous peoples of Manitoba represent six tribal/cultural or ethnic groupings: Lakota, Cree, Ojibway (Anishinabe), Oji-Cree, Dene, and Inuit. Most have signed historical Treaties with the Federal Government of Canada, and some reside on “un-ceded territories”, without formal Treaties. In contrast to healthcare funding which is provincial for non-Indigenous persons, funding for and administration of healthcare services to all Indigenous communities is Federal. Importantly, in Manitoba, and elsewhere in Canada, the Federal Indian Residential School System was in effect during much of the twentieth century, removing children from their families. This mass trauma has been linked to negative mental health outcomes that continue today through effects of intergenerational trauma [46].

## Call to Action: Implementing a Telemental Health Service

Prior to RNTS, Indigenous youth residing on reserve had very limited access to “specialized” mental health supports, with no established process for consultation or treatment. Indigenous citizens and healers have always strived to meet the needs and demands of their communities, but lacked a means to integrate with distant expertise available in the province. Only a small number of psychiatrists and psychologists were willing to provide services, requiring travel to their urban-based offices. The process to establish these services was often uncertain and could take months or years. By necessity, these needs were met on-reserve by primary care providers, including nurse practitioners and itinerant family physicians or pediatricians, or therapists where available. Youth presenting with acute mental health needs such as suicidality, or features of severe and persistent mental illness were sent via med-evac to a tertiary care center for emergency assessment, at a cost of up to $15,000 CAD per evacuation [47].

In 2010, after a striking number of deaths by suicide of Indigenous youth, a Provincial Suicide Prevention Network was established. Under this Network’s oversight, a pilot project was initiated with 4 Indigenous communities, whereby remote mental health services would be established for youth aged 5–17, using the provincial Telemedicine Network. Most Indigenous communities had telehealth equipment within their nursing stations or health centers, although the infrastructure and equipment in some locations was rudimentary. The RNTS guiding principles were to be sustainable, culturally safe, community-focused, accessible, collaborative, and serving high-risk youth, and aim for mental health promotion and capacity building. The treatment team included mental health therapists, a psychiatrist, and administrative support. A policy of “no wrong door” was adopted whereby youth could be referred from any concerned resource including nurses or physicians, educators, child welfare workers, addictions workers, police, and local or itinerant community mental health workers. Additionally, youth who had been transported to a tertiary care center could be referred to RNTS for follow-up care upon their discharge back to their community. With early evidence of success, and expanding awareness and support, the RNTS program grew to 15 remote Indigenous reserves by 2017, comprising hundreds of youth receiving services within their home communities.

Concurrent with local efforts to improve access to mental health services for Indigenous on-reserve Youth, a national process was underway to address widespread inequities in access to health services for the Indigenous population of Canada. A critical story of a Manitoban Indigenous boy, Jordan River Anderson, born with a disabling congenital condition who required complex care and could not receive it in his home community due to unresolved debates between Provincial and Federal Governments over financial responsibility, led to intense lobbying efforts among Indigenous representatives. In 2007, the Canadian House of Commons passed a law, entitled Jordan’s Principle, which outlined the responsibility of governments to provide healthcare to all Indigenous children, regardless of where they live, equal to what all other citizens would receive [6]. Subsequent lobbying and legislation ultimately led to the Federal Government providing funding for the implementation of Jordan’s Principle across Canada. In June 2017, the RNTS received funding to expand into all 63 Indigenous communities in Manitoba. With this growth, the RNTS treatment team expanded, and with additional Federal investment, the telehealth equipment and infrastructure are improving and many on-reserve school settings are becoming telehealth sites.

## Sustaining the Service: the Importance of Cultural Respect and Relationships

**Fig. 1:**
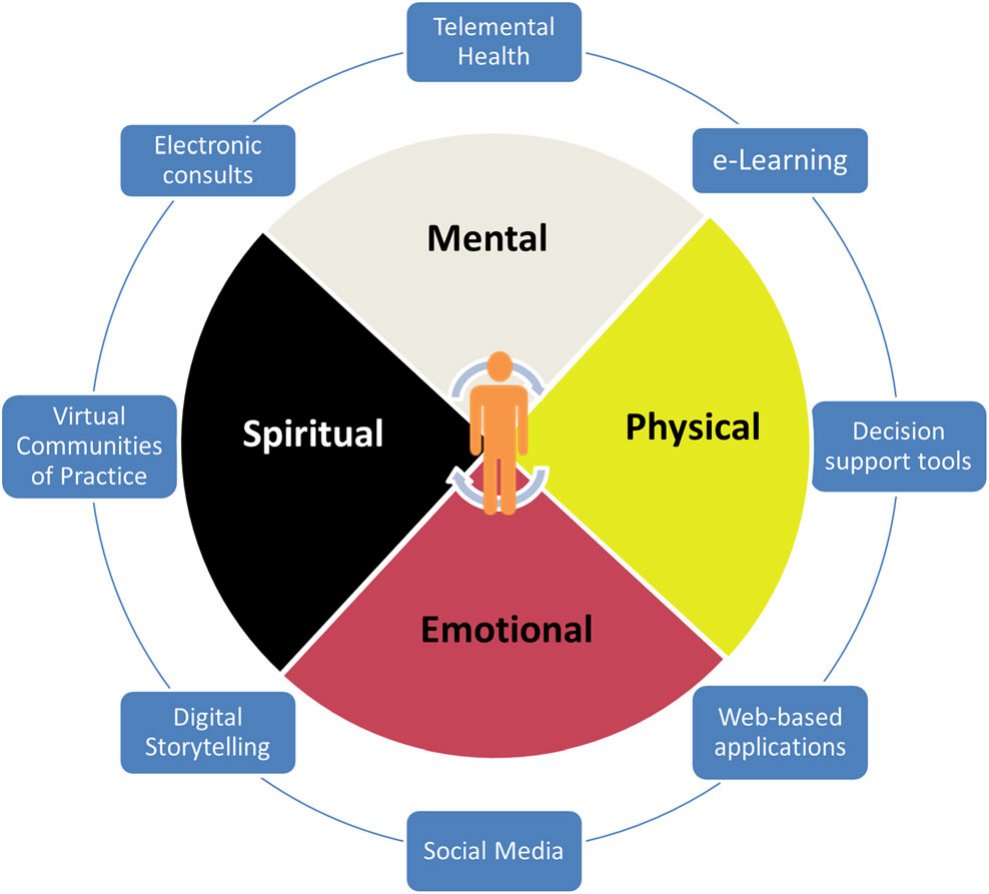
Digital health solutions for Indigenous mental well-being depicted around the Indigenous Medicine Wheel with the individual located at the center

Anthropologists, as well as Indigenous oral history, have determined that the Indigenous Peoples of North America have settled and thrived across the continent over the past 10,000 years, in thousands of communities, with hundreds of languages [48]. Much like Western society, Indigenous communities have unique paradigms for governance, justice, community social and cultural activities, arts, clothing and food creation and management, epistemology, education, engineering, spirituality, and healthcare, including mental health. Richard Katz describes in broad detail and depth the ancient, and at times globally coincidental healing “Psychologies” of Indigenous People from his international studies [49••]. He compares and contrasts the development of the Euro-centric psychologies, with the traditional ways of healing practiced in Indigenous societies. One fundamental unifying principle of Indigenous epistemology is humankind’s symbiotic relationship with the Earth and Heavens, practiced with reverence, and gratitude for the provisions of life available to us. Mental wellness is sought and achieved through experiencing and expanding one’s relationship with nature, and also practicing community ceremonies and rituals to both celebrate life and invoke health and healing. The traditional Indigenous “Medicine Wheel” illustrates a holistic approach to wellness, fundamental to a culturally appropriate model of healthcare delivery (Fig. 1). Laurence Kirmayer has written about the critical importance of Cultural literacy, respect, competency, and safety, in the delivery of health and mental health services [50]. Similarly, Indigenous leaders in Manitoba have repeatedly emphasized the absolute necessity of their people’s autonomy, self-governance, and the revitalization and widespread re-establishment of their traditional health and education practices.

Herein lies an inherent challenge for a Western and technology-based service: how do we best evolve our Western evidence-based mental health practices, while integrating traditional ways and knowledge, in an effective and respectful manner? Critical to the success of the RNTS program has been the pursuit and prioritization of traditional Indigenous teachings, and learning historical cultural ways of healing, simultaneously respecting and advocating for the availability and engagement in these practices for the population served. Findings from a recent study of eight Indigenous communities articulated best practices for culturally effective services: “You treat the whole person, within the family and within the community. You support the person in achieving balance in their emotional, physical, spiritual and mental health [51••].” Through regular connections with Indigenous communities and experts in Indigenous health, we have sought to understand, validate, and incorporate healing strategies to counter the oppressive forces which, through policies of cultural genocide invoked intergenerational trauma, familial dysfunction, wide-spread mental suffering, addictions, and socio-economic disadvantage relative to the colonizing Western majority of Canada. Over time, the RNTS program has evolved to supplement existing community-based resources with telemental health, and when able, integrating our specialty therapies into community models. Jordan’s Principle has provided more funding to allow RNTS team members to travel into communities, to meet, engage, and support the care recipients, which enhances therapeutic engagement and overall acceptance of the “Western” or “mainstream” service. Our team includes specialists in a wide variety of therapies, some of which lend themselves well to traditional ways. For example, horticultural therapy is a land-based approach to experiencing and learning fundamental principles which influence community health, self-esteem, and even food security. With shared learning, interesting and novel partnerships and approaches can be developed and fostered for improved community outcomes and programs which can be carried onward by local citizens.

## Conclusions

A variety of digital health solutions show promise in addressing the mental health needs of Indigenous peoples across the globe, and our RNTS program is local evidence of success. While digital health solutions seem to be acceptable to many Indigenous, there are also concerns—some similar to general population concerns about the rapid rise of digital healthcare, some unique to Indigenous people arising from lasting effects of colonialism alongside distinct cultural and health beliefs [14, 33••]. Indigenous youth and innovators are finding ways to utilize digital applications and social media, as a way to share ideas, promote positive behavior change, and generate healthy social networks. Non-Indigenous clinicians providing healthcare to Indigenous populations should strive to learn about the historical influences, and current preferences and values of their Indigenous care recipients [52]. Digital health solutions, like other healthcare services, need to be adapted and implemented with careful consideration of Indigenous values and local community needs (Fig. 1), and more rigorous evaluation is needed. Browne et al. [5•] outline 4 key service dimensions, and 10 practical intersecting strategies to optimize healthcare services for Indigenous peoples. Development and implementation of digital health solutions should also adhere to these guiding principles. In our experience, initiatives that are done thoughtfully, collaboratively, and respectfully, with a focus on building local capacity and integrating with community resources, can be most successful for improving Indigenous mental well-being.
